# Adherence to a food group-based dietary guideline and incidence of prediabetes and type 2 diabetes

**DOI:** 10.1007/s00394-019-02064-8

**Published:** 2019-07-24

**Authors:** Nicolette R. den Braver, Femke Rutters, Andrea L. J. Kortlever van der Spek, Dorina Ibi, Moniek Looman, Anouk Geelen, Petra Elders, Amber A. van der Heijden, Johannes Brug, Jeroen Lakerveld, Sabita S. Soedamah-Muthu, Joline W. J. Beulens

**Affiliations:** 1 Department of Epidemiology and Biostatistics, Amsterdam Public Health Research Institute, Amsterdam UMC, Vrije Universiteit Amsterdam, Amsterdam, The Netherlands; 2 Division of Human Nutrition, Wageningen University and Research, Wageningen, The Netherlands; 3 Department of General Practice and Elderly Care Medicine, Amsterdam Public Health Research Institute, Amsterdam UMC, Vrije Universiteit Amsterdam, Amsterdam, The Netherlands; 4 Faculty of Social and Behavioural Sciences, University of Amsterdam, Amsterdam, The Netherlands; 5 Dutch National Institute for Public Health and the Environment, Bilthoven, The Netherlands; 6 Julius Center for Health Sciences and Primary Care, University Medical Center Utrecht, Utrecht, The Netherlands; 7 Center of Research on Psychological and Somatic Disorders (CORPS), Department of Medical and Clinical Psychology, Tilburg University, Tilburg, The Netherlands; 8 Institute for Food, Nutrition and Health, University of Reading, Reading, RG6 6AR UK

**Keywords:** Type 2 diabetes, Intermediate hyperglycemia, Prediabetes, Dutch healthy diet index 2015, Dietary pattern

## Abstract

**Purpose:**

In this study, we investigated the association between adherence to the Dutch Healthy Diet index 2015 (DHD15-index) and incidence of prediabetes (preT2D) and Type 2 Diabetes (T2D) in a representative sample for the general Dutch population.

**Methods:**

Two prospective cohort studies, The Hoorn and The New Hoorn Study, were used for data analyses. In total, data from 2951 participants without diabetes at baseline (mean age 56.5 ± 7.5 years; 49.6% male) were harmonized. Baseline dietary intake was assessed with validated Food Frequency Questionnaires and adherence to the DHD15-index was calculated (range 0–130). PreT2D and T2D were classified according to the WHO criteria 2011. Poisson regression was used to estimate prevalence ratios between participant scores on the DHD15-index and preT2D and T2D, adjusted for follow-up duration, energy intake, socio-demographic, and lifestyle factors. Change in fasting plasma glucose levels (mmol/L) over follow-up was analysed using linear regression analyses, additionally adjusted for baseline value.

**Results:**

During a mean follow-up of 6.3 ± 0.7 years, 837 participants developed preT2D and 321 participants developed T2D. The highest adherence to the DHD15-index was significantly associated with lower T2D incidence [model 3, PR_T3vsT1_: 0.70 (0.53; 0.92), *p*_trend_ = 0.01]. The highest adherence to the DHD15-index pointed towards a lower incidence of preT2D [PR_T3vsT1_: 0.87 (0.74; 1.03), *p*_trend_ = 0.11]. Higher adherence to the DHD15-index was not associated with change in fasting plasma glucose levels [*β*_10point_: − 0.012 (− 0.034; 0.009)mmol/L].

**Conclusion:**

The present study showed that the highest compared to the lowest adherence to the DHD15-index was associated with a lower T2D incidence, and pointed towards a lower incidence of preT2D. These results support the benefits of adhering to the guidelines in T2D prevention.

**Electronic supplementary material:**

The online version of this article (10.1007/s00394-019-02064-8) contains supplementary material, which is available to authorized users.

## Introduction

An unhealthy diet is a modifiable risk factor for prediabetes (preT2D) and type 2 diabetes (T2D), and is, therefore, an important target behavior for prevention. To assess the role of diet in prevention of preT2D and T2D, many studies have focused on single nutrients or single foods, rather than dietary patterns. However, dietary patterns reflect mixtures of foods and nutrients and may have stronger effects on chronic disease than single food groups or nutrients [1, 2].

Dietary patterns that have been associated with T2D include the Alternative Healthy Eating Index (AHEI), the Mediterranean diet and the Dietary Approaches to Stop Hypertension (DASH). These measures generally assess the intake of recommended fruits, vegetables, whole grains, and nuts, and less recommended refined grains, red or processed meats, and sugar-sweetened beverages, and are associated with a decreased risk of T2D [3, 4]. According to a recent meta-analysis, a higher adherence to both the AHEI and DASH diet have been associated with a 20% lower risk of incident T2D [3], while The Mediterranean diet was associated with an 8% lower risk of preT2D [5] and with a 50% lower risk of T2D in a high-risk population [4]. However, these dietary patterns cannot be readily translated to the Dutch population, because dietary patterns and related guidelines may be country specific [6].

In 2015, the Dutch Health Council recommended new guidelines for a healthy diet for the Dutch general population. These guidelines focused on food groups rather than nutrients, partly to facilitate easier communication to and implementation by the general population. The aim of developing the guidelines was to prevent ten major chronic diseases, including T2D. The new Dutch dietary guidelines are evidence based and updated according to the most recent literature. This led to inclusion of novel food groups, such as tea and filtered coffee, which are individually associated with lower T2D incidence [7, 8]. Moreover, the guidelines are specified to foods frequently consumed by the Dutch population, such as whole-grain products and dairy. The similarity with earlier guidelines, such as the Alternate Healthy Eating Index (AHEI), Mediterranean diet score (MDS), and Dietary Approach to Stop Hypertension (DASH), is all recommend intake of more plant-based food products and a lower intake of animal foods. The main differences are that the Dutch guidelines now include novel food groups, compared to other guidelines, and that adherence is scored based on Dutch consumption values, whereas an AHEI is based on American consumption values or a DASH is based on quintiles in the study population. The Dutch Healthy Diet index of 2015 (DHD15-index) has been developed to assess the adherence to the Dutch dietary guidelines of 2015 [9], based on methods used in the Dutch Healthy Diet index of 2006.

Direct evidence on adherence to the DHD15-index and incident chronic diseases is still limited, with only one earlier study showing that high adherence to DHD15-index was associated with a lower risk of mortality [10]. Another study investigated the association between adherence to the Dutch guidelines 2015 and 10 chronic diseases in the Rotterdam Study, but did not use the DHD15-index [11]. This study showed that higher adherence to the Dutch dietary guidelines of 2015 was associated with a lower risk of mortality, stroke, chronic obstructive pulmonary disease, colorectal cancer, and depression, but not with T2D. The relation of the DHD15-index with preT2D, the precursor and reversible stage of T2D, has not yet been studied.

In our study, we therefore investigated the association between adherence to the DHD15-index and incidence of preT2D and T2D in the Hoorn Study and the New Hoorn Study.

## Methods

### Study design

We harmonized data from the Hoorn Study (HS) (*n* = 2484) and The New Hoorn Study (NHS) (*n* = 2807) cohorts. Both cohorts were similar in design, population characteristics, and questionnaires, and are described in detail elsewhere [12]. In short, participants from the general population were recruited between 1989 and 1992 for the HS and between 2006 and 2007 for the NHS. Inclusion criteria were age 50–75 years in the HS and 40–65 in the NHS at time of inclusion and ability to provide informed consent. The follow-up measurement was performed between 1996 to 1998 in the HS and between 2013 and 2015 in the NHS. For both studies, participants were included at the Diabetes Care Center in the city of Hoorn. Both cohorts were established to investigate the prevalence and risk factors of impaired glucose metabolism, diabetes, and diabetes-related complications in a predominately white population and whether increasing rates of longevity, physical inactivity, and obesity affected the prevalence and risk factors of disturbances in glucose metabolism.

**Fig. 1:**
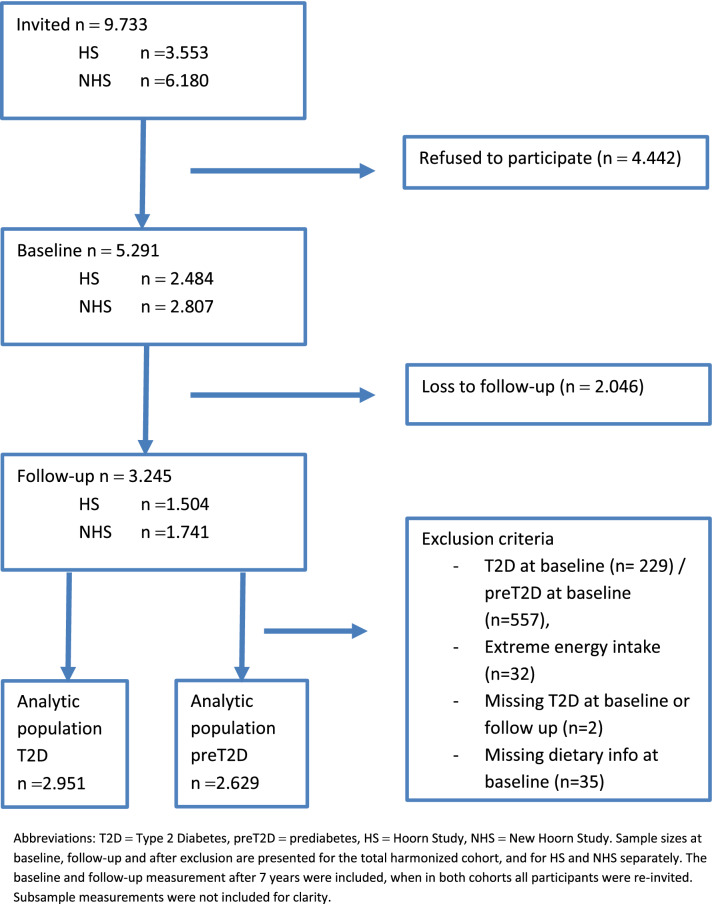
Flow chart of study population

For the present study, we excluded participants with preT2D and T2D at baseline for the analyses with outcome preT2D (*n*_preT2D_ = 557), and T2D at baseline was excluded for analyses with outcome T2D and fasting plasma glucose (*n*_T2D_ = 229). Exclusion of preT2D was based on fasting plasma glucose, 2 h glucose, and HbA1c, and exclusion of T2D was based on the same blood parameters, diagnosis by a general practitioner and medication user or medication use retrieved from dispensing labels. Other exclusion criteria were extreme energy intake (top and bottom 0.5%) (*n* = 32), and missing information on dietary intake (*n* = 35) or missing data on preT2D/T2D at baseline or follow-up (*n*_preT2D_ = 9, *n*_T2D_ = 2). After exclusion, the analytic sample consisted of 2951 participants for T2D analyses, and of 2629 for the preT2D analyses (Fig. 1).

### Dietary assessment

Dietary intake was assessed in both cohorts with a self-administered food frequency questionnaire (FFQ). In the HS, we used a 92-item FFQ and in the NHS a 104-item FFQ. Both FFQs were developed and validated by Wageningen University and Research [13–15]. The HS FFQ was validated against a dietary history in 74 men and women. The FFQ was valid for ranking individuals according to energy intake (*r* = 0.72) and macronutrients (*r* = 0.71) [14]. The NHS FFQ was validated against actual energy intake in controlled feeding trials, and was valid for ranking individuals according to energy intake (*r* = 0.83) [13]. Both questionnaires obtained information on frequency, portion size, and preparation. Intake per FFQ food item was calculated using the Dutch food composition table (NEVO) 1989/1990 for the HS FFQ and the NEVO 2006 for the NHS FFQ, and food items were then classified in the DHD15-index food groups. An FFQ food item was categorized in a DHD15 food group if the individual food items constructing the FFQ items attributed > 50% of the intake, as according to the Dutch National Food Consumption Survey. Because two different FFQs were used, intake per food group was stratified for cohort and reported in Supplementary File 1.

### Dutch Healthy diet index

Adherence to the Dutch Dietary Guidelines of 2015 was assessed with the DHD15-index, and details of this index are described elsewhere [9]. In short, the DHD15-index includes adequacy-, moderation-, optimum-, ratio-, and quality components. Each component is assigned a score between 0 (minimum score) and 10 (maximum score) (Supplementary File 2). First, adequacy components indicate that a higher intake receives a higher score, with a maximum score being allocated to an intake above a specified cutoff. The adequacy components in this index include vegetables, fruits, whole-grain products, unsalted nuts, legumes, fatty fish, and tea. Adequacy components were scored by dividing the intake by the cut-off value. Second, moderation components indicate that a lower intake receives a higher score, with a maximum score allocated to an intake below a specified threshold value. Moderation components include red meat, processed meat, alcohol, sugar-sweetened beverages (SSBs), alcohol, and sodium. Moderation components were scored by dividing intake by the threshold value and subtracting it from 10. Third, an optimum component indicates that there is an optimal intake range, receiving the maximum score. Dairy is the only optimum component in this index. This optimum component was scored by subtracting the cut-off value from the intake by the participant, and dividing it by the range of optimal intake. Fourth, ratio components were included for food products, where replacement of unhealthy with healthy products within the same food group is recommended, and where a higher healthy/unhealthy ratio receives a higher score, with a maximum score allocated to a ratio above a certain cutoff. The ratio components were for oils/solid fats and refined/whole-grain products. Ratio components were scored by subtracting the threshold value from the ratio of intake by the participant, and dividing it by the range between the threshold and cut-off value. Whole-grain products are an adequacy component as well as a ratio component, and, therefore, scores from 0 (minimum score) and 5 (maximum score) on each component, to form one total score for whole-grain products. Finally, quality components were product groups, where a specific type of product is recommended, and where such specific product receives a maximum score. The DHD15-index uses this quality component for coffee, where filtered coffee receives the maximum score, and unfiltered coffee the minimum score. In this study, no data were available about filtering of coffee (quality component) and sodium intake (moderation component) from the FFQs, so these components were excluded from the index. In addition, the FFQs did not distinguish between salted/unsalted nuts, so we used total intake of nuts as adequacy component. To calculate the DHD15-index, available component scores were summed per respondent, resulting in scores ranging from 0 (lowest adherence) to 130 (highest adherence). The index was used as a continuous variable as well as tertiles for further analyses.

### Outcome assessment

In both the HS and the NHS, two study visits were performed, where in both cohorts, all participants were re-invited after approximately 7 year follow-up. In both studies, participants came to the research center after an overnight fast (with the exception of water; from 8PM on the night before). During the visits, blood samples were drawn to determine fasting plasma glucose levels (FPG), 2-h glucose (2hG) levels after an 75-g oral glucose tolerance test (OGTT) and HbA1c levels, with the exception of the follow-up visit in the NHS, where no OGTT was conducted, and HbA1c was measured in capillary blood samples, using blood spot cards, a fasting blood sample was obtained.

FPG (mmol/L) and 2hG levels were determined, using the glucose dehydrogenase method in both cohorts (Merck, Darmstad, Germany). In the HS, HbA1c was determined by ion-exchange high-performance liquid chromatography with a Modular Diabetes Monitoring System (Bio-Rad, Veenendaal, The Netherlands). In the NHS at baseline, Hba1c levels were assessed using standardized reverse-phase cation-exchange chromatography (HA 8160 analyzer; Menarini, Florence, Italy). In the NHS follow-up, HbA1c levels were derived from blood spot cards, using thermo immunoturbidimetry according to a validated protocol [16].

PreT2D and T2D were defined according to the diagnostic criteria of the World Health Organization of 2006 [17]. This definition was complemented with HbA1c cut-off values based on the 2009 international expert committee report for preT2D [18], and on the WHO 2011 addendum on HbA1c cut-off values for T2D [19]. Participants were diagnosed as having preT2D with either FPG between 6.1 and 7.0 mmol/L, 2hG between 7.8 and 11.0 mmol/L, or HbA1c between 6.0 and 6.5%. Participants were diagnosed with T2D if either diagnosed by a general practitioner, used diabetes medication, and/or had an FPG ≥ 7.0 mmol/L, 2hG ≥ 11.1 mmol/L, or HbA1c ≥ 6.5%. All available measurements were included in T2D and preT2D definitions in baseline and follow-up definitions,

### Covariates

The baseline questionnaire for both cohorts included socio-demographic and lifestyle-related questions. Smoking status was self-reported and measured as being a current, former, or never smoker. Highest attained education was self-reported in eight categories, which we categorized into low (no education or primary school), middle (secondary education), and high (tertiary education). Self-reported physical activity was assessed using the SQUASH questionnaire [20, 21]. From this questionnaire, total physical activity (including light, moderate, and vigorous activities) in hours per week was derived.

Physical measurements were performed according to similar protocols in both cohorts. BMI was calculated as weight divided by height squared (kg/m^2^), measured with light clothing. Hypertension was defined as a mean systolic blood pressure ≥ 140 mmHg and diastolic blood pressure ≥ 90 mmHg over three measurements. Triglycerides were measured in fasting blood samples.

### Statistical analysis

Baseline characteristics were presented as proportions [*n* (%)], mean ± SD or median (IQR) as appropriate, in tertiles of adherence to the DHD15-index. Missing values in confounding variables (for 4.8% of participants, data on covariates were imputed, because they had 1 or more missing values. Percentages of missing variables ranged between 0.1% for hypertension and 3.5% for physical activity) were imputed using multiple imputation, with five imputed data sets according to predictive mean matching, and combined with Rubin’s rules [22].

We estimated Prevalence Ratio’s (PR) and 95% Confidence intervals with Poisson regression with robust variance because of the relatively high prevalence of the outcomes preT2D and T2D [23]. PRs were calculated for tertiles of adherence to the DHD15-index, and continuously per 10 points increase in the index, separately for T2D and preT2D. Linear trend across tertiles was assessed by including median values of each tertile as a continuous variable in the model. If a significant association was observed between the DHD15-index and T2D or preT2D, we investigated which food groups were driving the association by repeating the analyses, excluding each single food group from the index, and assessing attenuation of the association as compared to the full DHD15-index. Linear regression was performed to analyze change in fasting blood glucose (FPG) in participants without T2D and with an FPG at baseline and follow-up (*n* = 1603).

Three theory-based models were constructed and tested, all with preT2D or T2D as dependent variable and DHD15-index as independent variable. Model 1 included energy intake, follow-up duration, and cohort as possible confounders. Model 2 was additionally adjusted for age and sex, and model 3 was additionally adjusted for smoking, education, and physical activity. BMI was added separately in model 4 because of its role as a potential mediator, or otherwise confounding variable. The role of BMI as a potential mediator in this association was investigated by adding BMI at baseline to the final model, and assessing attenuation of the association. The linear regression models were also adjusted for baseline FPG level. The analyses were checked for effect modification by cohort, age, sex, and education, by including interaction terms in the model, and results were stratified in case of significant interaction (*p* < 0.05).

As a sensitivity analysis HbA1c was excluded from the follow-up definition of T2D and preT2D to assess the influence of using the capillary measurement in NHS. In addition, we assessed the association between adherence to the DHD15-index with change in and 2-h glucose (2hG) levels, in a subsample of participants of the HS with a baseline and follow-up 2hG measurement (*n* = 1294). A third set of sensitivity analyses was performed to assess the stability of the association due to the single dietary assessment. We excluded participants with self-reported cardiovascular disease (CVD) (*n* = 195), cancer (*n* = 27) or both (*n* = 4) at baseline, in a subsample of the HS (*n*_T2D_ = 1332, *n*_preT2D =_ 992), because these participants were most likely to change their dietary pattern during the study. No data on self-reported co-morbidities were yet available for the NHS cohort. All statistical analyses were performed using SPSS (version 22.0).

## Results

Table 1 summarizes the baseline characteristics of the total study population in tertiles of the DHD15-index. The mean age of the participants was 56.5 ± 7.5 years and 49.6% was male. The mean DHD15-index score was 69.5 ± 14.1 ranging from 0 to 109.3. Participants in the highest tertile of adherence to the guidelines (score 75.9–109.3) were on average older and more often non-smoker, had a higher education, were more physically active, had a lower BMI, and lower fasting blood glucose, as compared to participants in the lowest tertile (< 62.1) (Table 1). No significant interactions were found by cohort (*p*_T2D_ = 0.85, *p*_preT2D_ = 0.29), age (*p*_T2D_ = 0.47, *p*_preT2D_ = 0.20), sex (*p*_T2D_ = 0.94, *p*_preT2D_ = 0.43), or education (*p*_T2D_ = 0.91, *p*_preT2D_ = 0.84). These variables were treated as confounders in the analyses.

**Table 1:** Baseline characteristics (*n* = 2951) presented as mean ± SD, median (IQR), or *n* (%)

	*N*	Total population	DHD15-index tertiles		
			T1 (*n* = 982)	T2 (*n* = 986)	T3 (*n* = 983)
			< 63.7	63.7–74.9	> 74.9
Sex (male)	2951	1486 (49.6%)	511 (52.0%)	511 (51.8%)	464 (49.4%)
Age (years)	2951	56.5 ± 7.5	55.7 ± 7.0	57.0 ± 7.8	56.9 ± 7.5
BMI (kg/m^2^)	2947	26.1 ± 3.5	26.4 ± 3.5	26.3 ± 3.5	25.5 ± 3.3
Education					
Low	2914	421 (14.3%)	131 (13.3%)	159 (16.1)	131 (13.3%)
Middle		1699 (.6%)	587 (9.8%)	573 (8.1)	587 (9.8%)
High		794 (26.9%)	247 (25.2%)	244 (24.7)	247 (25.2%)
Smoking	2947				
Current		676 (22.9%)	309 (31.5%)	228 (21.2%)	139 (4.1%)
Previous (> 2 months ago)		1124 (38.1%)	381 (38.8%)	365 (37.0%)	378 (8.5%)
Never		1138 (38.6%)	284 (28.9%)	389 (39.5%)	465 (47.3%)
Cigarette years	2126	230.2 (497.4)	350.0 (576.5)	222.5 (485.8)	135.5 (389.5)
Physical activity, moderate intensity, min/week	2847	7.0 (8.2)	6.5 (8.0)	7.0 (8.5)	7.4 (8.2)
Triglycerides (mmol/L)	2951	1.2 (0.8)	1.3 (1.0)	1.2 (0.8)	1.1 (0.6)
Hypertension	2949	1015 (34.4%)	355 (36.2%)	347 (35.2%)	355 (36.2%)
Fasting glucose (mmol/L)	2944	5.4 ± 0.5	5.5 ± 0.5	5.4 ± 0.5	5.3 ± 0.5
Energy intake (kcal)	2951	2131.8 ± 595.8	2152.6 ± 631.9	2114.0 ± 605.0	2128.8 ± 692.0
DHD15-index score	2951	69.5 ± 14.1	53.8 ± 8.4	70.1 ± 3.5	84.5 ± 6.7
DHD15 components (g/day)					
Fruit	2945	176.0 (178.8)	89.1. (143.44)	203.0. (139.7)	231.7 (181.6)
Vegetables	2946	150.6 ± 71.5	132.0 ± 75.0	146.5 ± 80.1	173.2 ± 90.0
Whole sgrain	2941	11.6 (108.0)	1.4 (73.3)	22.6 (112.5)	67.1 (124.1)
Refined grain	2948	129.8 (197.1)	141.0 (189.2)	112.7 (190.8)	130.1 (207.4)
Legumes	2944	7.0 (14.0)	0.0 (7.7)	6.0 (12.1)	9.0 (14.2)
Nuts	2941	4.0 (9.8)	2.6 (9.0)	4.0 (9.8)	6.1 (13.5)
Cheese	2947	21.2 (25.7)	20.0 (26.3)	22.0 (26.2)	22.0 (25.3)
Dairy	2946	278.9 (272.3)	213.0 (281.5)	287.5 (285.3)	301.4 (220.6)
Lean fish	2945	8.2 (14.0)	7.0 (12.6)	8.0 (14.0)	9.3 (14.0)
Fatty fish	2945	2.9 (6.9)	0.6 (4.9)	3.0 (6.2)	3.7 (9.1)
Tea	2942	232.1 (294.7)	107.0 (229.9)	232.1 (286.0)	348.2 (267.9)
Liquid fat	2948	17.7 (27.5)	14.4 (25.7)	17.3 (27.7)	21.0 (28.0)
Solid fat	2582	17.6 (28.7)	20.0 (27.2)	19.7 (30.0)	13.0 (27.0)
Red meat	2945	34.6 ± 23.8	41.0 ± 27.2	33.2 ± 21.9	29.7 ± 20.4
Processed meat	2949	46.4 ± 33.1	54.6 ± 34.6	48.1 ± 33.1	36.5 ± 28.6
Sugar-sweetened beverages	2948	57.5 (160.4)	107.1 (222.6)	53.6 (150.6)	36.0 (114.0)
Alcohol	2946	7.3 (14.9)	12.8 (21.5)	6.8 (15.1)	5.0 (8.7)

During a mean follow-up of 6.3 ± 0.7 years, 321 out of 2951 participants developed T2D (10.9%). The highest adherence to the DHD15-index was associated with a 30% lower T2D incidence, compared to lowest adherence [model 3, PR_T3vsT1_: 0.70 (0.53; 0.92), *p*_trend_ = 0.01] (Table 2). Moderate adherence was not associated with T2D incidence [model 3, PR_T2vsT1_: 0.84 (0.64; 1.09)]. A ten-point increase in DHD-15-index pointed towards an association with a lower T2D incidence [model 3, PR_10point_: 0.95 (0.87; 1.03)]. The sensitivity analyses excluding HbA1c, resulted in 197 T2D cases, the effect size estimate was slightly stronger [model 3, PR_T3vsT1_: 0.63 (0.43; 0.90), *p*_trend_ = 0.01] (Supplementary File 3). In the sensitivity analyses excluding participants with CVD and/or cancer at baseline in a subsample of HS, the effect size estimate was stronger [model 3, PR_T3vsT2_: 0.53 (0.31; 0.87)] (Supplementary File 5). Adding BMI to the model attenuated the associations and rendered it non-significant, indicating that BMI partly mediates the association.

**Table 2:** Prevalence ratio’s (95% confidence interval) for the association between adherence to the DHD15-index and incidence of T2D (*n* = 2951) and preT2D (*n* = 2629)

T2D	T1 126/982	T2 109/986	T3 86/983	Continuous (per 10 point)	*P* for trend
Crude	1	0.86 (0.67; 1.11)	0.68 (0.52; 0.90)	0.94 (0.87; 1.01)	
Model 1	1	0.85 (0.66; 1.10)	0.70 (0.53; 0.92)	0.94 (0.87; 1.02)	0.01
Model 2	1	0.82 (0.64; 1.07)	0.68 (0.51; 0.89)	0.93 (0.86; 1.01)	0.005
Model 3	1	0.84 (0.64; 1.09)	0.70 (0.53; 0.92)	0.95 (0.87; 1.03)	0.01
Model 4	1	0.86 (0.65; 1.13)	0.76 (0.56; 1.02)	0.96 (0.88; 1.05)	0.04

PreT2D	T1 303/864	T2 267/863	T3 267/902	Continuous	*P* for trend
Crude	1	0.90 (0.76; 1.06)	0.87 (0.73; 1.02)	0.96 (0.91; 1.00)	
Model 1	1	0.91 (0.77; 1.08)	0.87 (0.74; 1.03)	0.96 (0.92; 1.01)	0.11
Model 2	1	0.91 (0.77; 1.07)	0.86 (0.73; 1.02)	0.96 (0.91; 1.00)	0.09
Model 3	1	0.91 (0.77; 1.07)	0.87 (0.74; 1.03)	0.96 (0.91; 1.01)	0.11
Model 4	1	0.92 (0.78; 1.09)	0.89 (0.75; 1.06)	0.97 (0.92; 1.01)	0.18

Model 1: Adjusted for total energy, FU time, cohort

Model 2: Additionally adjusted for age and gender

Model 3: Additionally adjusted for smoking, education, physical activity

Model 4: Addition of BMI

*T2D* type 2 Diabetes, *preT2D* prediabetes

During follow-up, 837 participants out of 2629 developed preT2D (32%). The highest adherence to the DHD15-index pointed towards a lower incidence of preT2D, compared to the lowest adherence [PR_T3vsT1_: 0.87 (0.74; 1.03), *p*_trend_ = 0.11]. (Table 2). A ten-point increase in DHD15-index pointed towards an association with a lower preT2D incidence [model 3, PR_10point_: 0.96 (0.91; 1.01)]. In sensitivity analyses, excluding HbA1c from the definition at follow-up, resulted in 526 preT2D cases and strengthened the association: the highest adherence to the DHD15-index showed a significant association with a 25% lower preT2D incidence [model 3, PR_T3vsT1_: 0.75 (0.60; 0.93), *p*_trend_ = 0.007] (Supplementary File 3). Exclusion of participants with CVD and/or cancer at baseline in a subsample of HS also resulted in a stronger effect size estimate [model 3, PR_T3vsT1_: 0.79 (0.59; 1.05)] (Supplementary File 5). Adding BMI to the model attenuated the association slightly, but remained significant in the sensitivity analyses, where HbA1c was excluded.

Individual exclusion of vegetables, fruits, nuts, tea, or SSBs from the DHD15-index attenuated the PRs for T2D incidence substantially and to non-significance (Table 3). Exclusion of grains, legumes, dairy, fish, fat ratio, red eat, processed meat, and alcohol did not alter the association.

**Table 3:** Prevalence ratio’s (95% confidence interval) for the association between DHD15-index in T2D, excluding single food groups

	T1 126/982	T2 109/986	T3 86/983	*P* for trend	Continuous (per 10 point)
DHD15-index^a^	1	0.84 (0.64; 1.09)	0.70 (0.53; 0.92)	0.01	0.95 (0.87; 1.03)
Excl. vegetables	1	0.97 (0.74; 1.29)	0.77 (0.57; 1.03)	0.08	0.93 (0.86; 1.02)
Excl. fruits	1	1.03 (0.79; 1.35)	0.75 (0.56; 1.01)	0.07	0.95 (0.87; 1.04)
Excl. grains	1	0.88 (0.67; 1.15)	0.72 (0.53; 0.97)	0.03	0.94 (0.87; 1.02)
Excl. legumes	1	0.91 (0.69; 1.19)	0.73 (0.54; 0.98)	0.04	0.94 (0.86; 1.03)
Excl. nuts	1	0.94 (0.71; 1.23)	0.78 (0.58; 1.05)	0.10	0.94 (0.87; 1.03)
Excl. dairy	1	0.91 (0.69; 1.19)	0.67 (0.50; 0.91)	0.01	0.93 (0.86; 1.02)
Excl. fish	1	0.85 (0.65; 1.12)	0.68 (0.51; 0.91)	0.01	0.93 (0.86; 1.02)
Excl. tea	1	1.12 (0.85; 1.47)	0.83 (0.62; 1.12)	0.23	0.96 (0.88; 1.05)
Excl. fat ratio	1	0.88 (0.67; 1.16)	0.73 (0.54; 0.99)	0.04	0.94 (0.86; 1.02)
Excl. red meat	1	0.93 (0.71; 1.22)	0.73 (0.54; 0.99)	0.04	0.96 (0.88; 1.04)
Excl. processed meat	1	0.91 (0.69; 1.19)	0.71 (0.53; 0.96)	0.03	0.95 (0.87; 1.03)
Excl. SSB	1	1.02 (0.77; 1.34)	0.86 (0.64; 1.15)	0.32	0.95 (0.87; 1.03)
Excl. alcohol	1	1.04 (0.80; 1.35)	0.65 (0.48; 0.88)	0.009	0.92 (0.85; 1.01)

^a^Model 3 presented, adjusted for total energy, FU time, cohort, age, sex, smoking, education, physical activity

Higher adherence to the DHD15-index was not associated with change in FPG levels over follow-up, in 1603 participants [*β*_10point_: − 0.012 (− 0.034; 0.009) mmol/L] (Table 4). In addition, higher adherence to the DHD15-index was not associated with change in 2-h glucose over follow-up, in 1294 HS participants [*β*_10point_: − 0.041 (− 0.120; 0.038) mmol/L] (Supplementary File 4).

**Table 4:** Association between adherence to the DHD15-index and change in fasting glucose (mmol/L) [beta (95% confidence interval)] (*n* = 1603)

	T1	T2	T3	Continuous (per 10 point)
Crude	Ref	− 0.010 (− 0.073; 0.053)	− 0.037 (0.95; 1.646)	− 0.020 (− 0.041; 0.001)
Model 1	Ref	− 0.010 (− 0.073; 0.054)	− 0.036 (− 0.101; 0.030)	− 0.019 (− 0.040; 0.002)
Model 2	Ref	− 0.010 (− 0.073; 0.053)	− 0.019 (-0.086; 0.049)	− 0.013 (− 0.035; 0.008)
Model 3	Ref	− 0.009 (− 0.073; 0.054)	− 0.016 (− 0.084; 0.052)	− 0.012 (− 0.034; 0.009)

Crude: Adjusted for baseline fasting glucose level

Model 1: Additionally adjusted for total energy, FU time, cohort

Model 2: Additionally adjusted for age and sex

Model 3: Additionally adjusted for smoking, education, physical activity

## Discussion

The present study showed that the highest compared to the lowest adherence tertile to the DHD15-index was associated with a lower T2D incidence, and pointed towards a lower incidence of preT2D. However, associations did not hold in the continuous analyses. In sensitivity analyses, excluding HbA1c from the definition of preT2D at follow-up, the highest adherence to the DHD15-index showed significant associations with a lower preT2D incidence, which was also observed in the increment analyses. The above-mentioned associations were attenuated after adjustments for BMI, which could possibly mean that BMI is a mediator in this association. The observed associations were mainly attributable to higher intake of fruits, tea, and lower intake of red meat, processed meat, and SSBs.

An earlier study investigating the adherence to the guidelines from 2015 and T2D incidence did not find an association with T2D incidence [hazard ratio: 1.01 (0.97; 1.06)] [11]. In that study, adherence to each food group was dichotomized (yes/no), rather than using a continuous adherence score [11], which is a less sensitive approach to assessing differences in guideline adherence between participants, and may explain some of the differences with our findings. Moreover, in our study, we used a slightly more sensitive measure of T2D, since we included OGTT and HbA1c measurements as well, while the previous study relied on fasting plasma glucose or non-fasting plasma glucose, and medication use. Using a selection of diagnostic criteria could have underestimated the number of T2D cases and attenuated the association in this previous study. Regarding effect sizes of dietary patterns on T2D, our study found similar effect sizes as the previous studies on other dietary indices. One meta-analyses including ten prospective cohort studies on the association between the Mediterranean diet score and T2D incidence found a pooled association of 0.77 (0.66–0.89) when comparing the highest to the lowest available centiles [24]. Another meta-analyses examined HEI (3 studies), AHEI (9 studies), and DASH score (7 studies) in relation to T2D, and found similar associations between high adherence and a lower risk of T2D, for all dietary indices [AHEI: 0.80 (0.74; 0.86), DASH: 0.80 (0.74; 0.86) HEI: 0.87 (0.82; 0.93)] [3].

In addition to the association with T2D, a higher adherence to the DHD15-index was also inversely associated to a lower risk of preT2D, but this association was not statistically significant, and no association with fasting plasma glucose was observed. This could be due to the different disturbances in glucose metabolism that each different measures of glycemic control may reflect. Two-hour glucose after an OGTT reflects muscle insulin sensitivity and thus the impaired uptake of glucose by muscles, whereas a measurement of fasting plasma glucose reflects hepatic insulin resistance resulting in an impaired suppression of glucose production (gluconeogenesis) [25, 26]. The previous studies have indeed indicated that lifestyle factors such as unhealthy diet, physical inactivity, and smoking are associated with higher 2-h glucose levels and thus peripheral insulin resistance, but not with fasting plasma glucose, hepatic insulin resistance [25]. Whereas genetic factors and family history were associated with a higher fasting plasma glucose [25]. This could explain why we found an association in the analyses, where 2-h glucose levels were included in the definition and not in the analyses including only fasting plasma glucose. When testing the changes in 2-h glucose in a subsample of our population (subsample of HS), we observed an inverse association with higher adherence to the DHD15-index, although effect sizes were small and non-significant. HbA1c is related to both biological processes and measurements have a higher reproducibility than fasting plasma glucose and 2-h glucose [27]; however, analyses of change in HbA1c was limited in our study because of capillary measurements in follow-up of the NHS. Our further analyses regarding specific food groups showed that a higher intake of vegetables, fruits, nuts, tea, and lower intake of SSBs were main drivers of the association with T2D, which is in line with earlier evidence that was used for the present dietary recommendations [28]. The exclusion of legumes, dairy, fatty fish, and fat ratio did not alter the association, and the Dutch dietary recommendations indeed do not claim that these food groups may reduce T2D risk [28]. Unexpectedly, we did not observe a change in the association between DHD15-index and T2D risk after excluding whole grains, red meat, and processed meat and alcohol. First, based on earlier scientific publications, whole grains were expected to be related to lower T2D and preT2D incidence [29, 30]. However, whole-grain intake was poorly measured in the NHS FFQ, because the way FFQ food items were grouped, which did not allow us to accurately distinguish whole grain from refined grain products. The HS FFQ did measure whole grains more adequately, but apparently did not provide enough power to observe an association. Second, for alcohol, the association becomes somewhat stronger after exclusion of alcohol from the index. The DHD15-index states that an intake less than 10 g/ day (1 consumption) obtains a maximum score, and an intake above 20 g (women) or 30 g (men)/day obtains a minimum score. This cut point is based on the association with increased risk of certain diseases, such as stroke, colorectal cancer, and breast cancer. However, an intake of 0–24 g (women) and 6–48 g (men) per day for men is associated with a 20% lower risk of T2D (10), which is above the cut point that the guideline classifies as positive (< 10 g). This may lead to some misclassification when scoring alcohol intake according to the DHD15-index in association with T2D, which may explain the associations to be somewhat stronger, when excluding it from the index. Third, the intake of red and processed meat was also low in our study population. According to the Dutch guidelines, an intake of more than 100 g/day of red meat, or more than 50 g per day of processed meat is associated with a higher risk of T2D [31]. The mean intake of red meat in our study population was below 100 g/day and the intake of processed meat was below 100 g/day (Table 1).

The Dutch health council compelled a shift from nutrient based to food based Dutch dietary guidelines. The guidelines were evidence-based focusing on the associations of each food group with ten major chronic diseases, but evidence on adherence to the DHD15-index and risk of chronic diseases was limited. This was the first study to investigate whether adherence to the DHD15-index is associated with lower incidence of preT2D and T2D. The results of this study support that adhering to the Dutch guidelines can contribute to the prevention of chronic diseases including T2D. The food group-based approach makes the guidelines easier to communicate to the general public, and can, therefore, be more easily incorporated in interventions. Future studies should focus on incorporating these guidelines in interventions, as it is difficult for the general population to adhere to dietary guidelines [32].

Strengths of the present study were the harmonization of the two Dutch cohorts, which were similar in design and increased our study power, the longitudinal design with 7 years of follow-up, and the use of the DHD15-index. However, certain limitations need to be addressed. A limitation of the present study was the exclusion of the DHD15-index components coffee and sodium, because the FFQs were not designed to assess this. However, inclusion of these components would probably not have altered our results. Drinking filtered coffee is associated with a lower T2D risk [7], but is included as a dichotomous score [9], and since most people consume filtered coffee, this will assign a maximum score to majority of participants and is unlikely to alter the results [7]. Moreover, sodium intake is, to our knowledge, not associated with T2D, so exclusion of this component is also not likely to have affected our results [28]. Second, the single dietary measurement at baseline could be considered as a limitation, although dietary patterns have shown to be reasonably stable [33]. To address this limitation, we performed a sensitivity analysis in a subsample excluding those who were likely to change their dietary pattern (exclusion of CVD and/or cancer at baseline), which resulted in stronger effect estimates indicating that the associations may in reality be stronger than can be shown in this study. Third, the missing OGTT and venous HbA1c at follow-up in the NHS limited inference on HbA1c changes over time and the use of these markers in the definition of preT2D and T2D. The exclusion of HbA1c from the follow-up definition of (pre)T2D resulted in strengthening of the associations, indicating an overestimation of preT2D and T2D cases when including HbA1c. This is in line with the literature, showing that HbA1c is a somewhat less sensitive marker of T2D [27] and capillary HbA1c samples also tend to be higher in HbA1c levels than in venous samples [34].

In conclusion, the highest adherence to the DHD15-index seems to be associated with a lower risk of T2D incidence, compared to the lowest adherence, but this did not hold in the increment analyses. Associations were rendered non-significant with additional adjustment of BMI. In addition, the highest adherence may be associated with a lower incidence of preT2D. A higher intake of vegetables, fruits, nuts, tea, and lower intake of SSBs was mainly responsible for the observed associations. This study confirms the benefits of adhering to the dietary guidelines for the prevention of T2D, one of the 10 diseases that the guideline targeted.

## Electronic supplementary material

Below is the link to the electronic supplementary material.

Supplementary File 1: food group intake (grams/day) stratified by cohort (DOCX 14 kb)

Supplementary File 2: Scoring of the DHD15-index (DOCX 15 kb)

Supplementary File 3: Sensitivity analyses without HbA1c follow-up, Prevalence Ratio’s (95% confidence interval) for the association between adherence to the DHD15 and incidence of T2D (n= 2951) and preT2D (n= 2629). (DOCX 14 kb)

Supplementary File 4: Sensitivity analyses association between adherence to the DHD15-index and change in 2-hour glucose (mmol/L) (beta (95% confidence interval)) (n=1294). (DOCX 12 kb)

Supplementary File 5: Sensitivity analyses for association between adherence to the DHD15-index and incidence of T2D (n=1332) and preT2D (n=992), in a subsample of HS where cases of CVD and/or cancer at baseline were excluded. (DOCX 13 kb)
