# Correction to: Adherence to a food group-based dietary guideline and incidence of prediabetes and type 2 diabetes

**DOI:** 10.1007/s00394-020-02402-1

**Published:** 2021-01-05

**Authors:** Nicolette R.den Braver, Femke Rutters, Andrea L. J. Kortlever van der Spek, Dorina Ibi, Moniek Looman, Anouk Geelen, Petra Elders, Amber A. van der Heijden, Johannes Brug, Jeroen Lakerveld, Sabita S. Soedamah-Muthu, Joline W. J. Beulens

**Affiliations:** 1 Department of Epidemiology and Biostatistics, UMC, Amsterdam Public Health Research Institute, Vrije Universiteit Amsterdam, Amsterdam, The Netherlands; 2 Division of Human Nutrition, Wageningen University and Research, Wageningen, The Netherlands; 3 Department of General Practice and Elderly Care Medicine, UMC, Amsterdam Public Health Research Institute, Vrije Universiteit Amsterdam, Amsterdam, The Netherlands; 4 Faculty of Social and Behavioural Sciences, University of Amsterdam, Amsterdam, The Netherlands; 5 Dutch National Institute for Public Health and the Environment, Bilthoven, The Netherlands; 6 Julius Center for Health Sciences and Primary Care, University Medical Center Utrecht, Utrecht, The Netherlands; 7 Center of Research On Psychological and Somatic Disorders (CORPS), Department of Medical and Clinical Psychology, Tilburg University, Tilburg, The Netherlands; 8 Institute for Food, Nutrition and Health, University of Reading, Reading, RG6 6AR UK

## Correction to: European Journal of Nutrition (2020) 59:2159–2169 10.1007/s00394-019-02064-8

The original version of this article unfortunately contained a mistake. The correct information is given below.

In abstracts, last sentence of the “Result” section should read: Higher adherence to the DHD15-index was not associated with change in fasting plasma glucose levels [*β*_10point_: − 0.010 (− 0.29; 0.008)mmol/L].

In “Statistical analysis” section, last sentence of the second paragraph should read: Linear regression was performed to analyze change in fasting blood glucose (FPG) in participants without T2D and with an FPG at baseline and follow-up (*n* = 2937).

In “Results” section, first sentence of the fifth paragraph should read: Higher adherence to the DHD15-index was not associated with change in FPG levels over follow-up, in 2937 participants [*β*_10point_: − 0.010 (− 0.029; 0.008) mmol/L] (Table 4).

The corrected Table 4 is given in the following page.

**Table 4:** Association between adherence to the DHD15-index and change in fasting glucose (mmol/L) [beta (95% confidence interval)] (*n* = 2937)

	T1	T2	T3	Continuous (per 10 poin)
Crude	Ref	0.044 (− 0.009; 0.097)	− 0.038 (− 0.091; 0.015)	− 0.009 (− 0.027; 0.009)
Model 1	Ref	0.033 (− 0.019; 0.086)	− **0.063 (**− **0.116; **− **0.010)**	− 0.013 (− 0.030; 0.005)
Model 2	Ref	0.033 (− 0.019; 0.086)	− **0.060 (**− **0.113; **− **0.007)**	− 0.012 (− 0.029; 0.006)
Model 3	Ref	0.034 (− 0.020; 0.087)	− 0.053 (− 0.108; 0.002)	− 0.010 (− 0.029; 0.008)

Crude: Adjusted for baseline fasting glucose level

Model 1: Additionally adjusted for total energy, FU time, cohort

Model 2: Additionally adjusted for age and sex

Model 3: Additionally adjusted for smoking, education, physical activity

