# Supplementary material for: Adherence to a food group-based dietary guideline and incidence of prediabetes and type 2 diabetes
Source: Eur J Nutr. 2019 Jul 24;59(5):2159–69. doi: 10.1007/s00394-019-02064-8 (PMC7351860; doi:10.1007/s00394-019-02064-8)
Supplement: Supplementary file 1 — food group intake (grams/day) stratified by cohort (DOCX 14 kb) [file 394_2019_2064_MOESM1_ESM.docx]

|  | **Hoorn Study (n = 1332)** | **New Hoorn Study (n = 1619)** |
| --- | --- | --- |
| DHD15 index | 70.3 ± 13.0 | 68.8 ± 14.9 |
| DHD15 components (g/day): |  |  |
| - Fruit | 226.5 (184.0) | 158.4 (151.5) |
| - Vegetables | 108.1 ± 46.1 | 185.7 ± 91.2 |
| - Wholegrain | 116.5 (83.0) | 0 (5.8) |
| - Refined grain | 15.0 (38.0) | 210.3 (115.5) |
| - Legumes | 7.0 (11.0) | 7.7 (19.2) |
| - Nuts | 2.0 (9.0) | 6.1 (8.1) |
| - Cheese | 23.0 (23.0) | 18.7 (28.4) |
| - Dairy | 417.8 ± 262.7 | 230.6 ± 150.7 |
| - Lean fish | 7.0 (18.0) | 8.8 (8.9) |
| - Fatty fish | 0.0 (6.0) | 3.7 (7.4) |
| - Tea | 250.0 (375.0) | 232.1 (303.6) |
| - Liquid fat | 9.0 (25.0) | 23.1 (25.9) |
| - Solid fat | 27.0 (30.0) | 8.2 (17.2) |
| - Red meat | 25.0 (28.8) | 38.7 (26.4) |
| - Processed meat | 61.4 ± 36.9 | 34.1 ± 23.1 |
| - Sugar sweetened beverages | 21.0 (116.8) | 96.4 (168.1) |
| - Alcohol | 5.0 (14.6) | 8.8 (18.3) |
