# Supplementary material for: Adherence to a food group-based dietary guideline and incidence of prediabetes and type 2 diabetes
Source: Eur J Nutr. 2019 Jul 24;59(5):2159–69. doi: 10.1007/s00394-019-02064-8 (PMC7351860; doi:10.1007/s00394-019-02064-8)
Supplement: Supplementary file 2 — Scoring of the DHD15-index (DOCX 15 kb) [file 394_2019_2064_MOESM2_ESM.docx]

| **Componentˆ** | | **Dutch dietary guidelines 2015** | **Minimum score = 0 (threshold level)** | **Maximum score = 10**  **(cut-off level)** |
| --- | --- | --- | --- | --- |
| *Adequacy components** | | | | |
| 1. | Vegetables | Eat at least 200 grams of vegetables a day. | 0 g/day | ≥ 200 g/day |
| 2. | Fruit | Eat at least 200 grams of fruit a day. | 0 g/day | ≥ 200 g/day |
| 3. | Wholegrain products | Eat at least 90 g of wholegrain products daily | 0 g/day | ≥ 90 g/day |
| 4. | Nuts˚ | Eat at least 15 grams of unsalted nuts a day. | 0 g/day | ≥ 15 g/day |
| 5. | Legumes | Eat legumes each week. | 0 g/day | ≥ 10 g/day |
| 6. | Fatty fish | Eat one time fatty fish each week. | 0 g/day | ≥ 15 g/day |
| 7. | Tea | Drink three cups of tea a day. | 0 ml/day | ≥ 450 ml/day |
| *Moderation components** | | | | |
| 8. | Red meat | Limit the consumption of red meat. | ≥ 100 g/day | ≤ 45 g/day |
| 9. | Processed meat | Limit the consumption of processed meat. | ≥ 50 g/day | 0 g/day |
| 10. | SSBs  juices | Limit the consumption of SSBs and fruit juices. | ≥ 250 ml/day | 0 ml/day |
| 11. | Alcohol | Limit the consumption of alcohol each day. | Women ≥ 20 g/day (ethanol)  Men ≥ 30 g/day (ethanol) | Women ≤ 10 g/day (ethanol)  Men ≤ 10 g/day (ethanol) |
| *Optimum component** | | | | |
| 12. | Dairy products | Take a few portions of dairy products each day, including yoghurt and milk. | 0 g/day OR ≥ 750 g/day | 300 - 450 g/day |
| *Ratio component** | | | | |
| 13. | Fats and oils | Replace hard margarine, hard cooking fats, and butter (hard fats/oils) by soft margarines, liquid cooking fats, and vegetable oils (soft fats/oils). | No intake of soft fats and oils OR  Ratio soft fats and oils/solid fats ≤ 0.7 | No intake of solid fats OR ratio soft fats and oils/solid fats ≥ 13 |
| 3. | Wholegrain products | Replace refined cereal products by wholegrain products | No intake of wholegrain products OR Ratio wholegrain/refined grain ≤ 0.6 | No intake of refined products OR ratio wholegrain/refined grain ≥ 11 |

SSB = sugar sweetened beverage, ˆCoffee and sodium were not presented in this table, because they could not be included in the present study, ˚No distinction could be made between salted and unsalted nuts, so total intake of nuts was included*calculation of components: adequacy score= $\frac{intake}{cut-off value}*10$, moderation score=$10-\frac{intake}{threshold value}*10$, optimum score = $10- \frac{10*(intake-cut-off value)}{optimum range}*10$, ratio component = $10* \frac{ratio of intake-threshold value}{ratio range}$
