# Supplementary material for: Adherence to a food group-based dietary guideline and incidence of prediabetes and type 2 diabetes
Source: Eur J Nutr. 2019 Jul 24;59(5):2159–69. doi: 10.1007/s00394-019-02064-8 (PMC7351860; doi:10.1007/s00394-019-02064-8)
Supplement: Supplementary file 3 — Sensitivity analyses without HbA1c follow-up, Prevalence Ratio’s (95% confidence interval) for the association between adherence to the DHD15 and incidence of T2D (n= 2951) and preT2D (n= 2629). (DOCX 14 kb) [file 394_2019_2064_MOESM3_ESM.docx]

| T2D | T1 | | T2 | | T3 | | Continuous (per 10 point) | | P for trend | |  |
| --- | --- | --- | --- | --- | --- | --- | --- | --- | --- | --- | --- |
|  | 70/984 | | 72/985 | | 55/982 | |  | |  | |  |
| Crude | 1 | | 0.97 (0.70; 1.34) | | 0.65 (0.46; 0.94) | | 0.91 (0.83; 1.01) | |  | |  |
| Model 1 | 1 | | 0.87 (0.63; 1.21) | | 0.63 (0.44; 0.90) | | 0.90 (0.81; 0.99) | | 0.01 | |  |
| Model 2 | 1 | | 0.84 (0.61; 1.17) | | 0.61 (0.42; 0.87) | | 0.89 (0.80; 0.98) | | 0.007 | |  |
| Model 3 | 1 | | 0.85 (0.62; 1.18) | | 0.63 (0.43; 0.90) | | 0.90 (0.81; 1.00) | | 0.01 | |  |
| Model 4 | 1 | | 0.89 (0.66; 1.20) | | 0.71 (0.49; 1.02) | | 0.93 (0.83; 1.03) | | 0.07 | |  |
| PreT2D | | **T1** | | **T2** | | **T3** | | **Continuous** | | **P for trend** | |
|  | | 198/864 | | 163/863 | | 165/902 | |  | |  | |
| Crude | | 1 | | 0.80 (0.65; 0.99) | | 0.77 (0.62; 0.95) | | 0.92 (0.87; 0.98) | |  | |
| Model 1 | | 1 | | 0.77 (0.63; 0.95) | | 0.75 (0.61; 0.92) | | 0.91 (0.86; 0.97) | | 0.006 | |
| Model 2 | | 1 | | 0.76 (0.62; 0.94) | | 0.74 (0.60; 0.91) | | 0.91 (0.86; 0.97) | | 0.005 | |
| Model 3 | | 1 | | 0.77 (0.62; 0.94) | | 0.75 (0.60; 0.93) | | 0.91 (0.86; 0.97) | | 0.007 | |
| Model 4 | | 1 | | 0.78 (0.63; 0.96) | | 0.78 (0.63; 0.97) | | 0.92 (0.87; 0.98) | | 0.02 | |

T2D= Type 2 Diabetes, preT2D= prediabetes

Model 1 total energy, FU time, cohort

Model 2 age and gender

Model 3 smoking, education, physical activity

Model 4 Addition of BMI
