# Supplementary material for: Adherence to a food group-based dietary guideline and incidence of prediabetes and type 2 diabetes
Source: Eur J Nutr. 2019 Jul 24;59(5):2159–69. doi: 10.1007/s00394-019-02064-8 (PMC7351860; doi:10.1007/s00394-019-02064-8)
Supplement: Supplementary file 4 — Sensitivity analyses association between adherence to the DHD15-index and change in 2-hour glucose (mmol/L) (beta (95% confidence interval)) (n=1294). (DOCX 12 kb) [file 394_2019_2064_MOESM4_ESM.docx]

|  | T1 | T2 | T3 | Continuous |
| --- | --- | --- | --- | --- |
| Crude | ref | -0.026 (-0.237; 0.185) | -0.032 (-0.239; 0.175) | -0.035 (-0.113; 0.043) |
| Model 1 | ref | -0.026 (-0.237; 0.185) | -0.041 (-0.247; 0.166) | -0.039 (-0.117; 0.039) |
| Model 2 | ref | -0.016 (-0.226; 0.193) | -0.081 (-0.290; 0.127) | -0.056 (-0.135; 0.023) |
| Model 3 | ref | -0.31 (-0.239; 0.177) | -0.047 (-0.255; 0.161) | -0.041 (-0.120; 0.038) |

Crude: Adjusted for baseline 2-hour glucose levels

Model 1: Additionally adjusted for total energy, FU time, cohort

Model 2: Additionally adjusted for age and sex

Model 3: Additionally adjusted for smoking, education, physical activity
