# Supplementary material for: Adherence to a food group-based dietary guideline and incidence of prediabetes and type 2 diabetes
Source: Eur J Nutr. 2019 Jul 24;59(5):2159–69. doi: 10.1007/s00394-019-02064-8 (PMC7351860; doi:10.1007/s00394-019-02064-8)
Supplement: Supplementary file 5 — Sensitivity analyses for association between adherence to the DHD15-index and incidence of T2D (n=1332) and preT2D (n=992), in a subsample of HS where cases of CVD and/or cancer at baseline were excluded. (DOCX 13 kb) [file 394_2019_2064_MOESM5_ESM.docx]

| T2D | T1  37/320 | T2  46/398 | T3  24/388 | Continuous (per 10 point) | P for trend | |
| --- | --- | --- | --- | --- | --- | --- |
| Crude | 1 | 1.00 (0.65; 1.54) | 0.54 (0.32; 0.89) |  |  | |
| Model 1 | 1 | 0.94 (0.61; 1.44) | 0.51 (0.31; 0.85) | 0.88 (0.76; 1.02) | 0.01 | |
| Model 2 | 1 | 0.91 (0.59; 1.41) | 0.51 (0.30; 0.86) | 0.86 (0.74; 0.99) | 0.01 | |
| Model 3 | 1 | 0.91 (0.59; 1.42) | 0.53 (0.31; 0.87) | 0.86 (0.74; 1.00) | 0.02 | |
| Model 4 | 1 | 0.92 (0.60; 1.44) | 0.55 (0.32; 0.94) | 0.87 (0.75; 1.02) | 0.03 | |
| PreT2D | **T1** | **T2** | **T3** | **Continuous** | **P for trend** |  |
|  | 47/292 | 44/341 | 35/359 |  |  |  |
| Crude | 1 | 0.92 (0.69; 1.22) | 0.82 (0.62; 1.10) | 0.95 (0.87; 1.03) |  |  |
| Model 1 | 1 | 0.92 (0.69; 1.22) | 0.82 (0.61; 1.09) | 0.94 (0.87; 1.03) | 0.16 |  |
| Model 2 | 1 | 0.91 (0.68; 1.20) | 0.80 (0.60; 1.07) | 0.94 (0.86; 1.02) | 0.14 |  |
| Model 3 | 1 | 0.89 (0.67; 1.19) | 0.79 (0.59; 1.05) | 0.93 (0.86; 1.01) | 0.12 |  |
| Model 4 | 1 | 0.92 (0.69; 1.22) | 0.83 (0.62; 1.11) | 0.94 (0.90; 0.99) | 0.21 |  |

T2D= Type 2 Diabetes, preT2D= prediabetes

Model 1: Adjusted for total energy, FU time, cohort

Model 2: Additionally adjusted for age and gender

Model 3: Additionally adjusted for smoking, education, physical activity

Model 4: Addition of BMI
